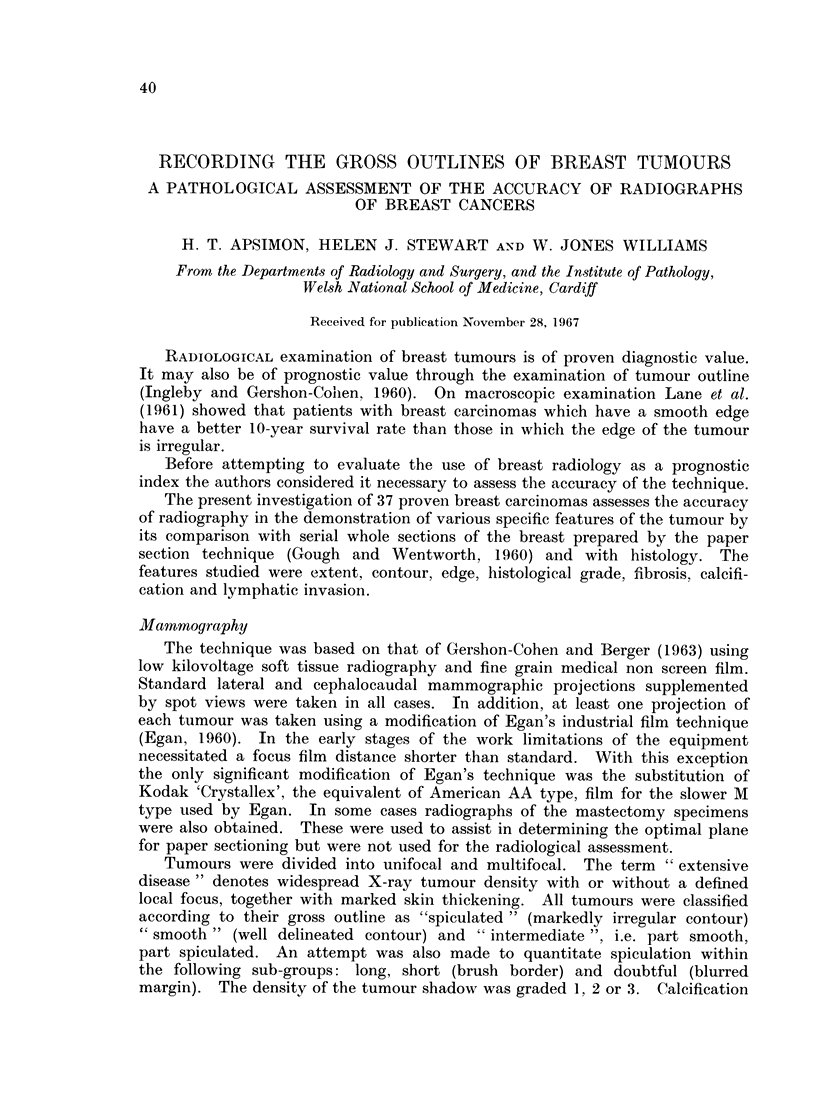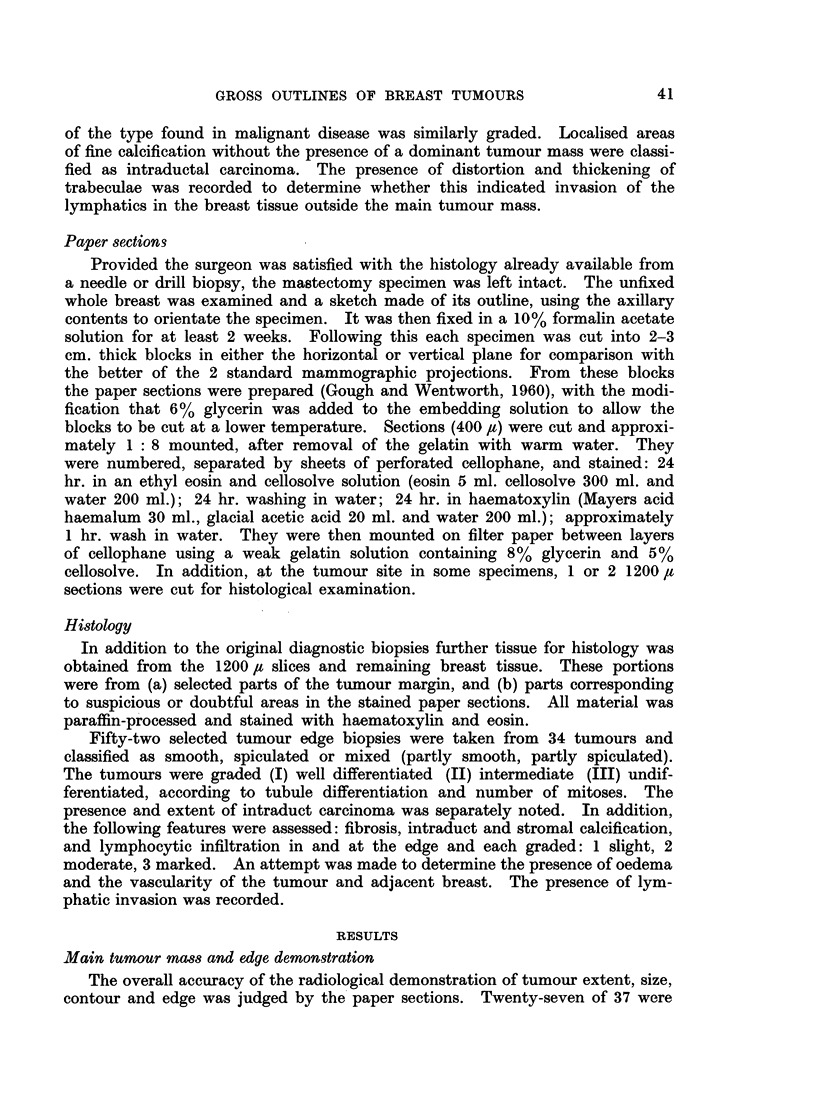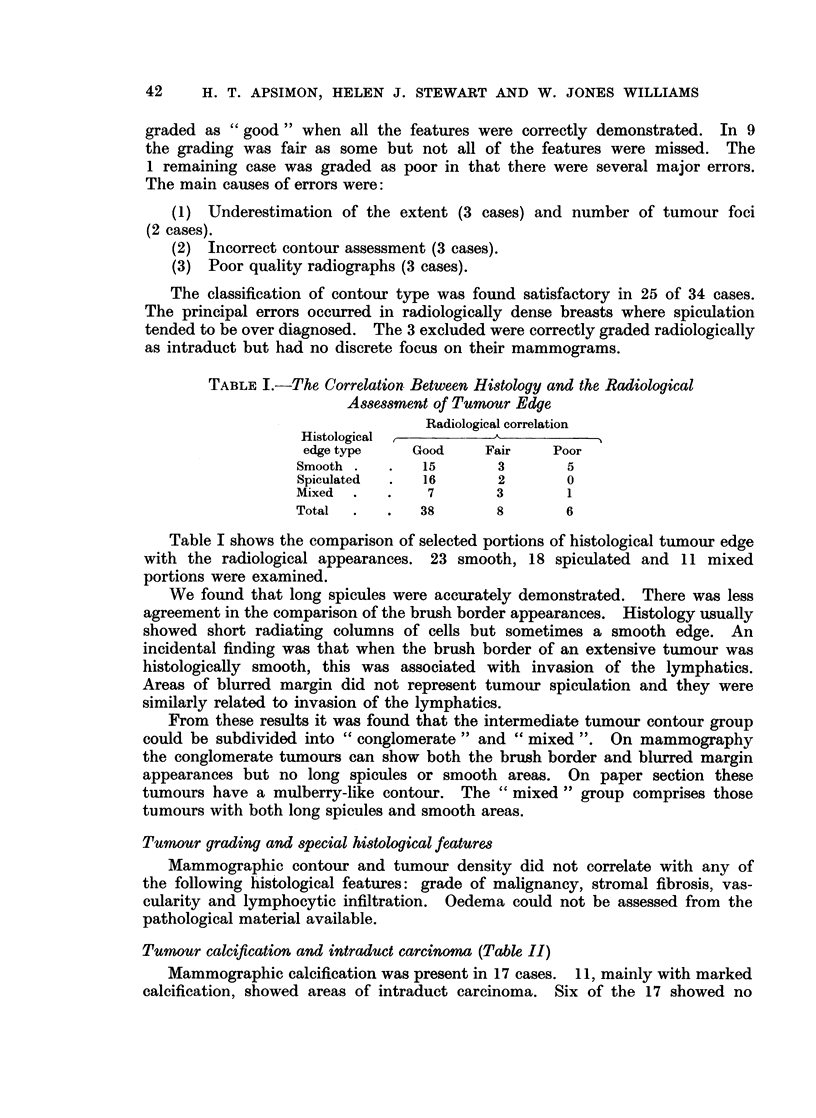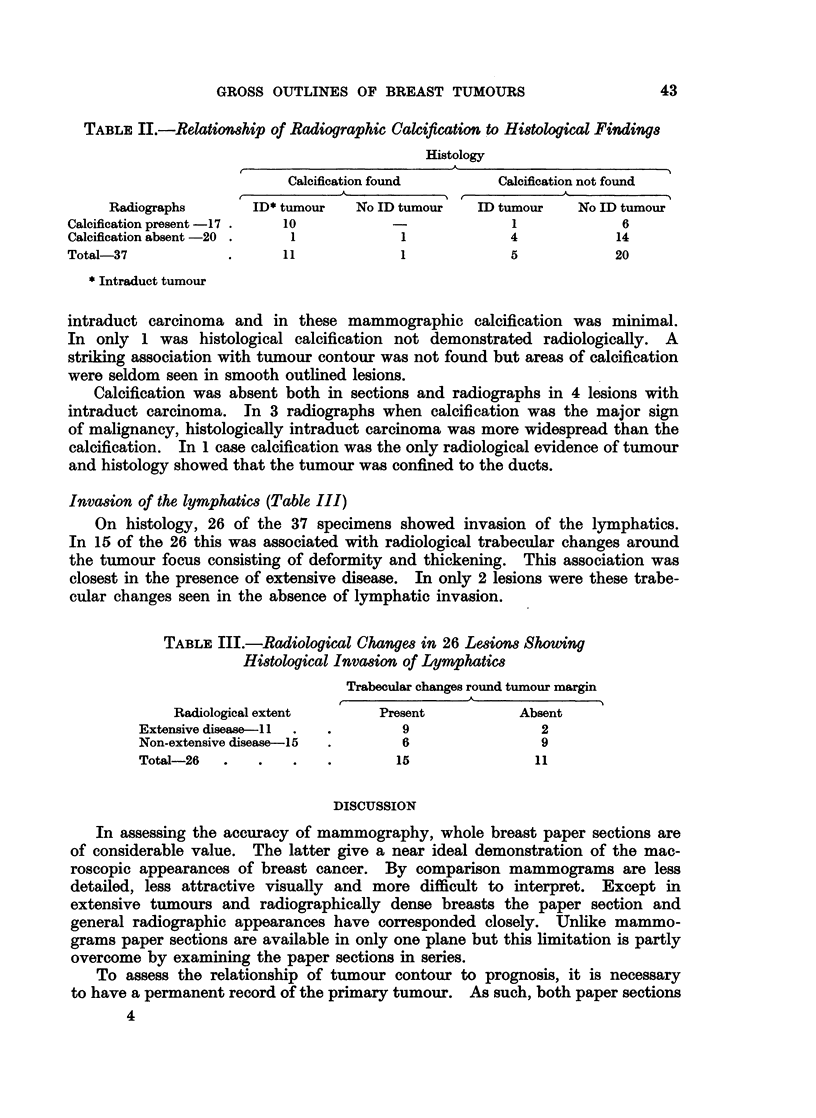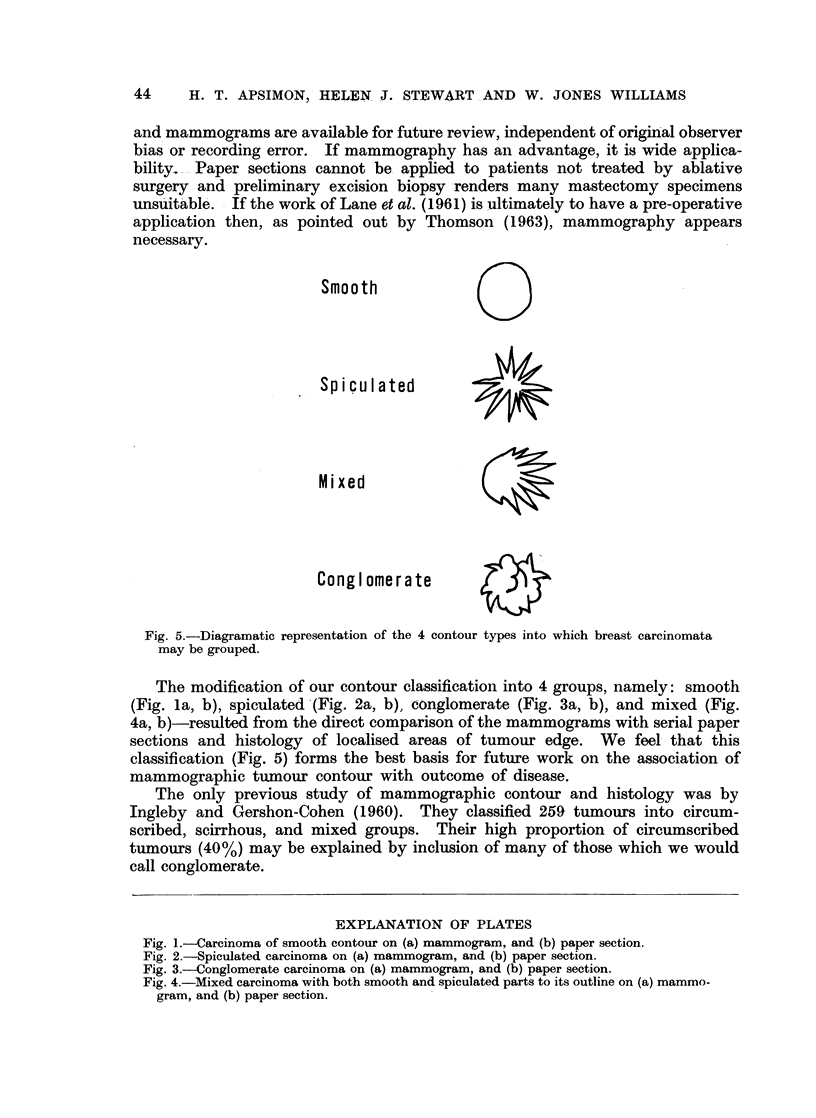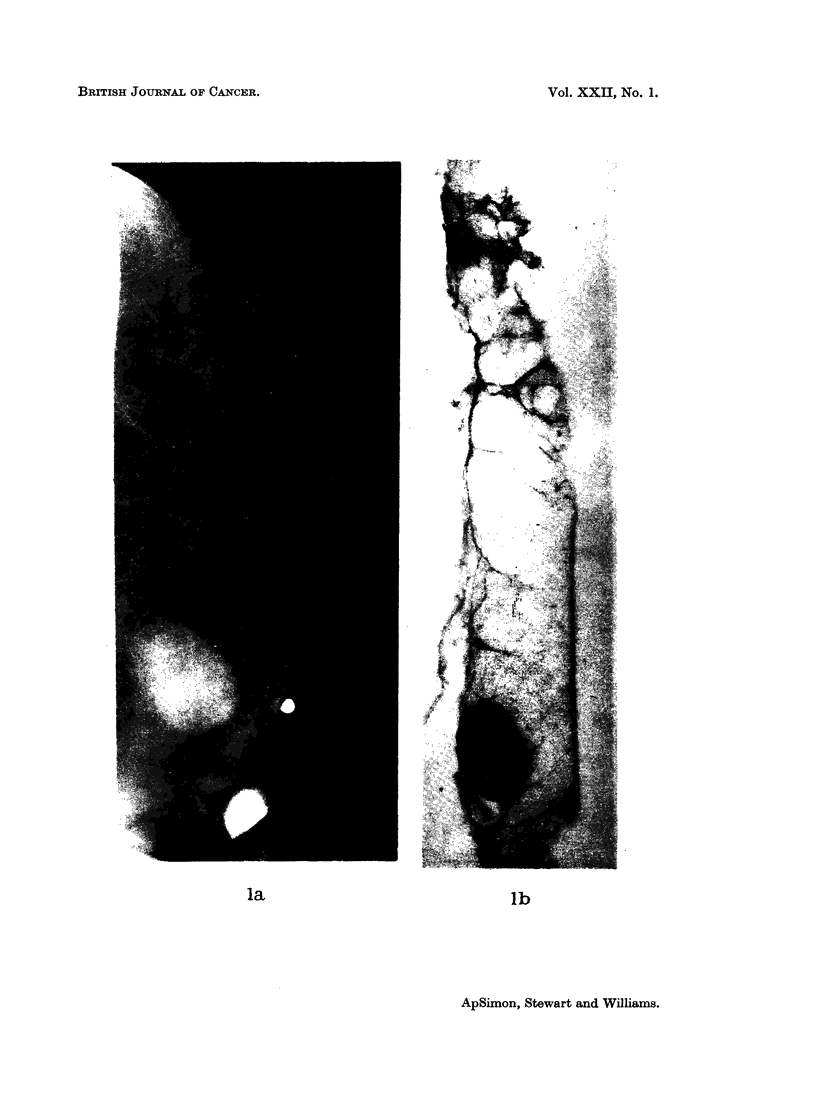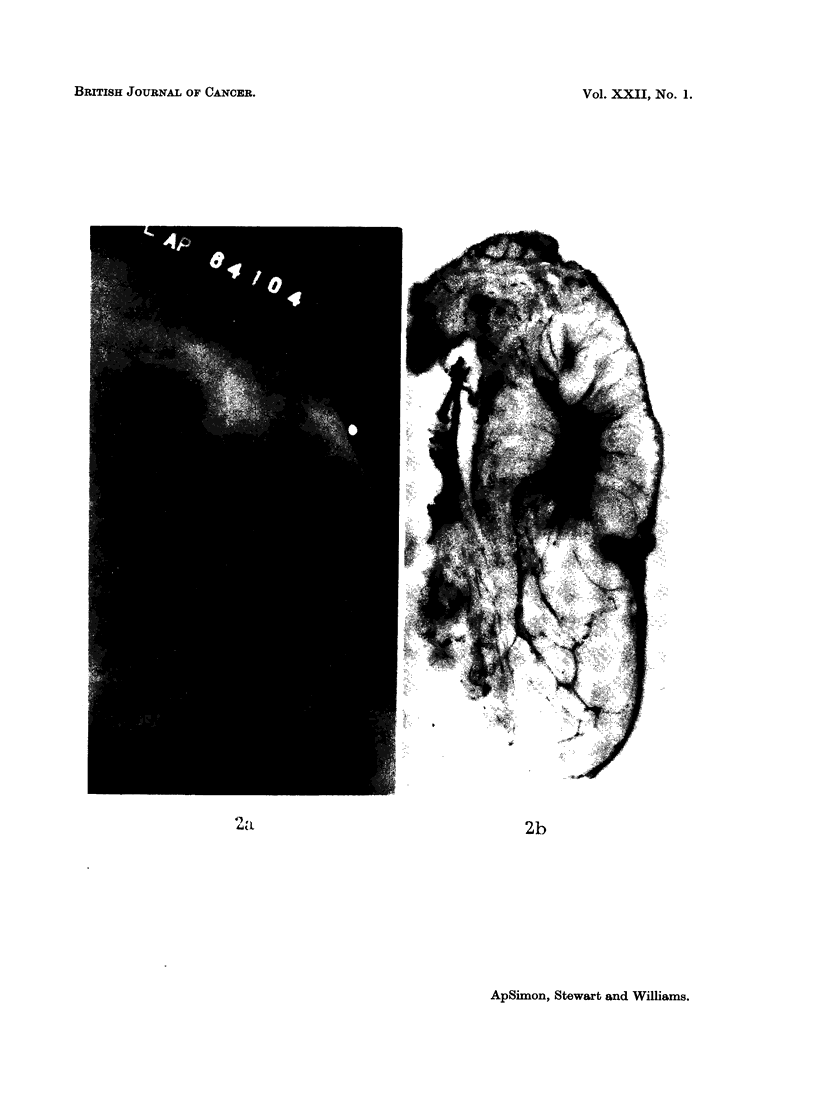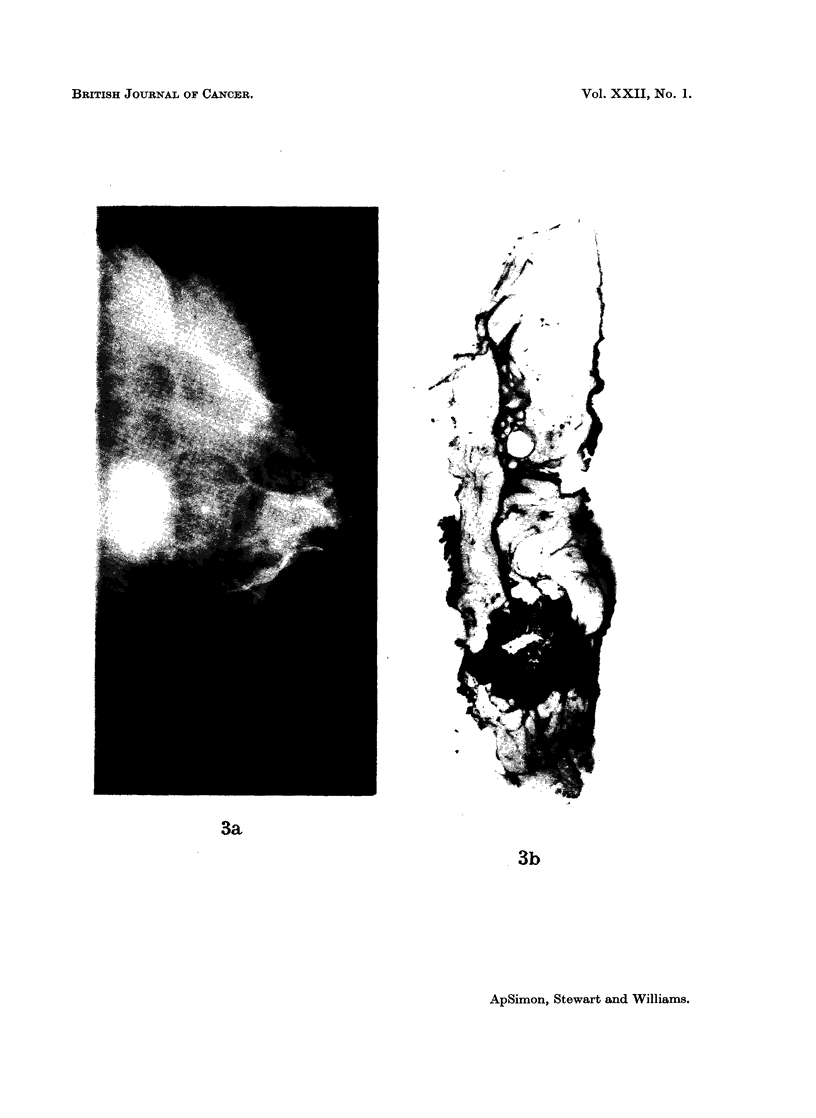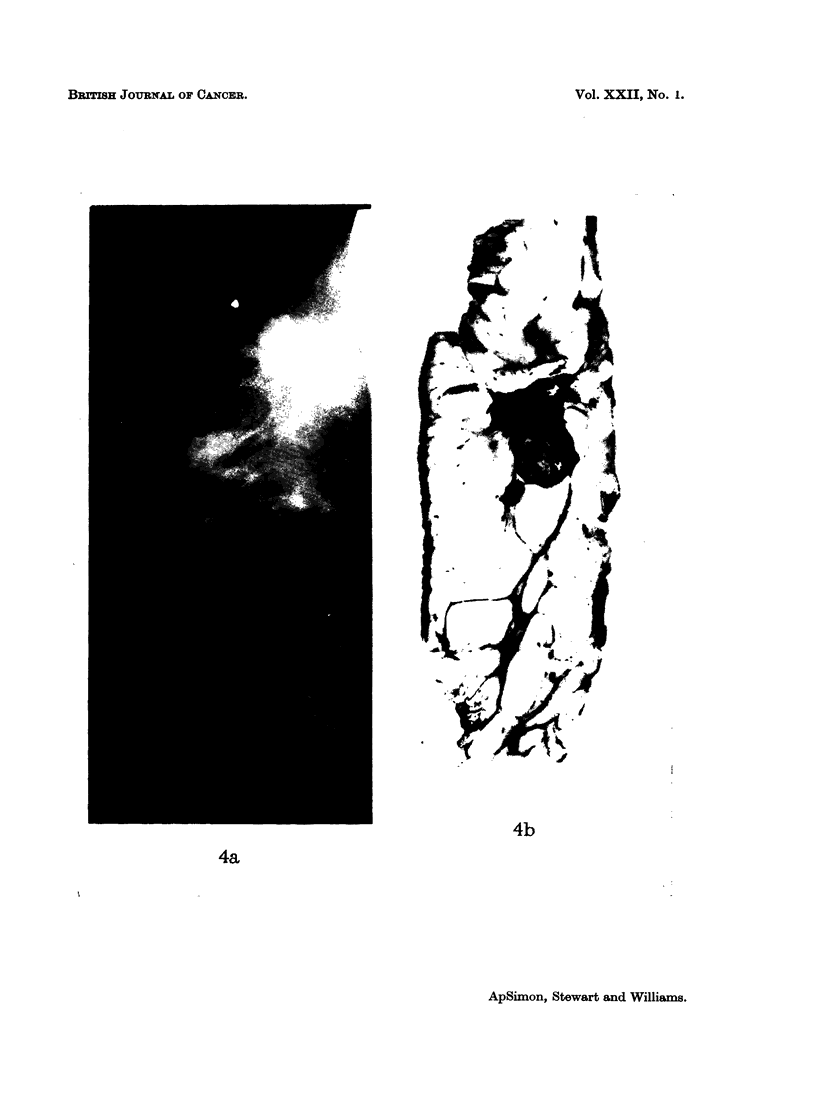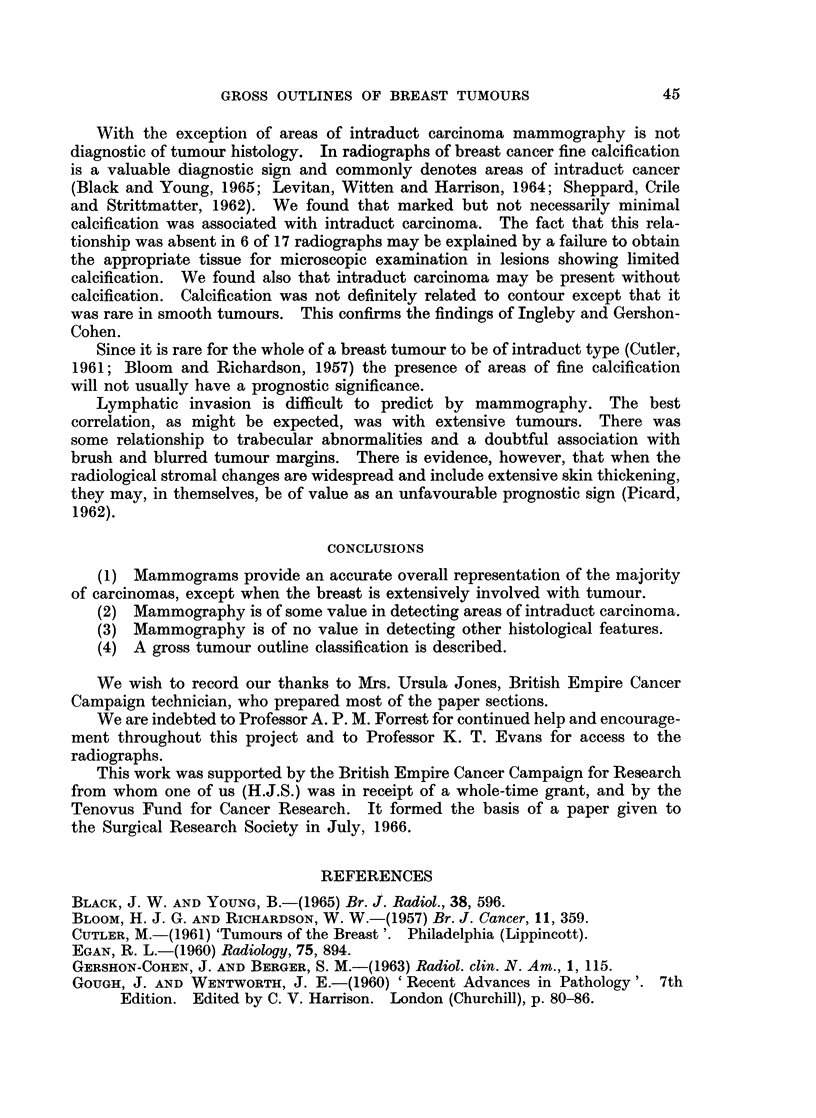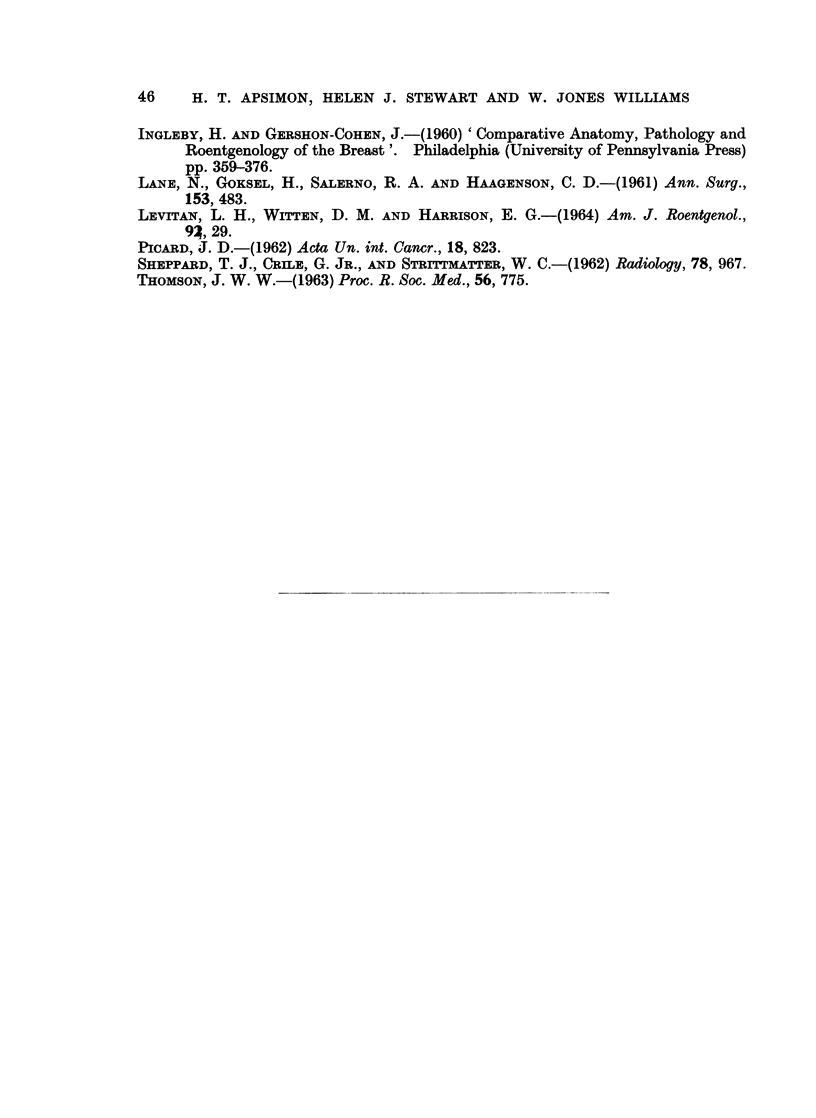# Recording the gross outlines of breast tumours. A pathological assessment of the accuracy of radiographs of breast cancers.

**DOI:** 10.1038/bjc.1968.6

**Published:** 1968-03

**Authors:** H. T. Apsimon, H. J. Stewart, W. J. Williams

## Abstract

**Images:**


### RECORDING THE GROSS OUTLINES OF BREAST TUMOURS

### A PATHOLOGICAL ASSESSMENT OF THE ACCURACY OF RADIOGRAPHS OF BREAST CANCERS

H. T. APSIMON, HELEN J. STEWART AND W. JONES WILLIAMS

*From the Departments of Radiology and Surgery, and the Institute of Pathology, Welsh National School of Medicine, Cardiff*

Received for publication November 28, 1967

RADIOLOGICAL examination of breast tumours is of proven diagnostic value. It may also be of prognostic value through the examination of tumour outline (Ingleby and Gershon-Cohen, 1960). On macroscopic examination Lane *et al.* (1961) showed that patients with breast carcinomas which have a smooth edge have a better 10-year survival rate than those in which the edge of the tumour is irregular.

Before attempting to evaluate the use of breast radiology as a prognostic index the authors considered it necessary to assess the accuracy of the technique.

The present investigation of 37 proven breast carcinomas assesses the accuracy of radiography in the demonstration of various specific features of the tumour by its comparison with serial whole sections of the breast prepared by the paper section technique (Gough and Wentworth, 1960) and with histology. The features studied were extent, contour, edge, histological grade, fibrosis, calcification and lymphatic invasion.

*Mammography*

The technique was based on that of Gershon-Cohen and Berger (1963) using low kilovoltage soft tissue radiography and fine grain medical non screen film. Standard lateral and cephalocaudal mammographic projections supplemented by spot views were taken in all cases. In addition, at least one projection of each tumour was taken using a modification of Egan's industrial film technique (Egan, 1960). In the early stages of the work limitations of the equipment necessitated a focus film distance shorter than standard. With this exception the only significant modification of Egan's technique was the substitution of Kodak 'Crystallex', the equivalent of American AA type, film for the slower M type used by Egan. In some cases radiographs of the mastectomy specimens were also obtained. These were used to assist in determining the optimal plane for paper sectioning but were not used for the radiological assessment.

Tumours were divided into unifocal and multifocal. The term " extensive disease " denotes widespread X-ray tumour density with or without a defined local focus, together with marked skin thickening. All tumours were classified according to their gross outline as "spiculated " (markedly irregular contour) " smooth " (well delineated contour) and " intermediate ", i.e. part smooth, part spiculated. An attempt was also made to quantitate spiculation within the following sub-groups: long, short (brush border) and doubtful (blurred margin). The density of the tumour shadow was graded 1, 2 or 3. Calcification of the type found in malignant disease was similarly graded. Localised areas of fine calcification without the presence of a dominant tumour mass were classified as intraductal carcinoma. The presence of distortion and thickening of trabeculae was recorded to determine whether this indicated invasion of the lymphatics in the breast tissue outside the main tumour mass.

*Paper sections*

Provided the surgeon was satisfied with the histology already available from a needle or drill biopsy, the mastectomy specimen was left intact. The unfixed whole breast was examined and a sketch made of its outline, using the axillary contents to orientate the specimen. It was then fixed in a 10% formalin acetate solution for at least 2 weeks. Following this each specimen was cut into 2–3 cm. thick blocks in either the horizontal or vertical plane for comparison with the better of the 2 standard mammographic projections. From these blocks the paper sections were prepared (Gough and Wentworth, 1960), with the modification that 6% glycerin was added to the embedding solution to allow the blocks to be cut at a lower temperature. Sections (400 $\mu$) were cut and approximately 1 : 8 mounted, after removal of the gelatin with warm water. They were numbered, separated by sheets of perforated cellophane, and stained: 24 hr. in an ethyl eosin and cellosolve solution (eosin 5 ml. cellosolve 300 ml. and water 200 ml.); 24 hr. washing in water; 24 hr. in haematoxylin (Mayers acid haemalum 30 ml., glacial acetic acid 20 ml. and water 200 ml.); approximately 1 hr. wash in water. They were then mounted on filter paper between layers of cellophane using a weak gelatin solution containing 8% glycerin and 5% cellosolve. In addition, at the tumour site in some specimens, 1 or 2 1200 $\mu$ sections were cut for histological examination.

*Histology*

In addition to the original diagnostic biopsies further tissue for histology was obtained from the 1200 $\mu$ slices and remaining breast tissue. These portions were from (a) selected parts of the tumour margin, and (b) parts corresponding to suspicious or doubtful areas in the stained paper sections. All material was paraffin-processed and stained with haematoxylin and eosin.

Fifty-two selected tumour edge biopsies were taken from 34 tumours and classified as smooth, spiculated or mixed (partly smooth, partly spiculated). The tumours were graded (I) well differentiated (II) intermediate (III) undifferentiated, according to tubule differentiation and number of mitoses. The presence and extent of intraduct carcinoma was separately noted. In addition, the following features were assessed: fibrosis, intraduct and stromal calcification, and lymphocytic infiltration in and at the edge and each graded: 1 slight, 2 moderate, 3 marked. An attempt was made to determine the presence of oedema and the vascularity of the tumour and adjacent breast. The presence of lymphatic invasion was recorded.

### RESULTS

*Main tumour mass and edge demonstration*

The overall accuracy of the radiological demonstration of tumour extent, size, contour and edge was judged by the paper sections. Twenty-seven of 37 were graded as " good " when all the features were correctly demonstrated. In 9 the grading was fair as some but not all of the features were missed. The 1 remaining case was graded as poor in that there were several major errors. The main causes of errors were:

(1) Underestimation of the extent (3 cases) and number of tumour foci (2 cases).
(2) Incorrect contour assessment (3 cases).
(3) Poor quality radiographs (3 cases).

The classification of contour type was found satisfactory in 25 of 34 cases. The principal errors occurred in radiologically dense breasts where spiculation tended to be over diagnosed. The 3 excluded were correctly graded radiologically as intraduct but had no discrete focus on their mammograms.

TABLE I.—*The Correlation Between Histology and the Radiological Assessment of Tumour Edge*

| Histological edge type | Radiological correlation | | |
|---|---|---|---|
| | Good | Fair | Poor |
| Smooth | 15 | 3 | 5 |
| Spiculated | 16 | 2 | 0 |
| Mixed | 7 | 3 | 1 |
| Total | 38 | 8 | 6 |

Table I shows the comparison of selected portions of histological tumour edge with the radiological appearances. 23 smooth, 18 spiculated and 11 mixed portions were examined.

We found that long spicules were accurately demonstrated. There was less agreement in the comparison of the brush border appearances. Histology usually showed short radiating columns of cells but sometimes a smooth edge. An incidental finding was that when the brush border of an extensive tumour was histologically smooth, this was associated with invasion of the lymphatics. Areas of blurred margin did not represent tumour spiculation and they were similarly related to invasion of the lymphatics.

From these results it was found that the intermediate tumour contour group could be subdivided into " conglomerate " and " mixed ". On mammography the conglomerate tumours can show both the brush border and blurred margin appearances but no long spicules or smooth areas. On paper section these tumours have a mulberry-like contour. The " mixed " group comprises those tumours with both long spicules and smooth areas.

*Tumour grading and special histological features*

Mammographic contour and tumour density did not correlate with any of the following histological features: grade of malignancy, stromal fibrosis, vascularity and lymphocytic infiltration. Oedema could not be assessed from the pathological material available.

*Tumour calcification and intraduct carcinoma (Table II)*

Mammographic calcification was present in 17 cases. 11, mainly with marked calcification, showed areas of intraduct carcinoma. Six of the 17 showed no

TABLE II.—*Relationship of Radiographic Calcification to Histological Findings*

| | Histology | | | |
|---|---|---|---|---|
| | Calcification found | | Calcification not found | |
| Radiographs | ID* tumour | No ID tumour | ID tumour | No ID tumour |
| Calcification present —17 . | 10 | — | 1 | 6 |
| Calcification absent —20 . | 1 | 1 | 4 | 14 |
| Total—37 . | 11 | 1 | 5 | 20 |

* Intraduct tumour intraduct carcinoma and in these mammographic calcification was minimal. In only 1 was histological calcification not demonstrated radiologically. A striking association with tumour contour was not found but areas of calcification were seldom seen in smooth outlined lesions.

Calcification was absent both in sections and radiographs in 4 lesions with intraduct carcinoma. In 3 radiographs when calcification was the major sign of malignancy, histologically intraduct carcinoma was more widespread than the calcification. In 1 case calcification was the only radiological evidence of tumour and histology showed that the tumour was confined to the ducts.

*Invasion of the lymphatics* (*Table III*)

On histology, 26 of the 37 specimens showed invasion of the lymphatics. In 15 of the 26 this was associated with radiological trabecular changes around the tumour focus consisting of deformity and thickening. This association was closest in the presence of extensive disease. In only 2 lesions were these trabecular changes seen in the absence of lymphatic invasion.

TABLE III.—*Radiological Changes in 26 Lesions Showing Histological Invasion of Lymphatics*

| | Trabecular changes round tumour margin | |
|---|---|---|
| Radiological extent | Present | Absent |
| Extensive disease—11 . . | 9 | 2 |
| Non-extensive disease—15 . | 6 | 9 |
| Total—26 . . . . | 15 | 11 |

### DISCUSSION

In assessing the accuracy of mammography, whole breast paper sections are of considerable value. The latter give a near ideal demonstration of the macroscopic appearances of breast cancer. By comparison mammograms are less detailed, less attractive visually and more difficult to interpret. Except in extensive tumours and radiographically dense breasts the paper section and general radiographic appearances have corresponded closely. Unlike mammograms paper sections are available in only one plane but this limitation is partly overcome by examining the paper sections in series.

To assess the relationship of tumour contour to prognosis, it is necessary to have a permanent record of the primary tumour. As such, both paper sections

4

and mammograms are available for future review, independent of original observer bias or recording error. If mammography has an advantage, it is wide applicability. Paper sections cannot be applied to patients not treated by ablative surgery and preliminary excision biopsy renders many mastectomy specimens unsuitable. If the work of Lane *et al.* (1961) is ultimately to have a pre-operative application then, as pointed out by Thomson (1963), mammography appears necessary.

Fig. 5.—Diagramatic representation of the 4 contour types into which breast carcinomata may be grouped.

The modification of our contour classification into 4 groups, namely: smooth (Fig. 1a, b), spiculated (Fig. 2a, b), conglomerate (Fig. 3a, b), and mixed (Fig. 4a, b)—resulted from the direct comparison of the mammograms with serial paper sections and histology of localised areas of tumour edge. We feel that this classification (Fig. 5) forms the best basis for future work on the association of mammographic tumour contour with outcome of disease.

The only previous study of mammographic contour and histology was by Ingleby and Gershon-Cohen (1960). They classified 259 tumours into circumscribed, scirrhous, and mixed groups. Their high proportion of circumscribed tumours (40%) may be explained by inclusion of many of those which we would call conglomerate.

---

### EXPLANATION OF PLATES

Fig. 1.—Carcinoma of smooth contour on (a) mammogram, and (b) paper section.
Fig. 2.—Spiculated carcinoma on (a) mammogram, and (b) paper section.
Fig. 3.—Conglomerate carcinoma on (a) mammogram, and (b) paper section.
Fig. 4.—Mixed carcinoma with both smooth and spiculated parts to its outline on (a) mammogram, and (b) paper section.

1a 1b

2a

2b

ApSimon, Stewart and Williams.

3a

3b

4a

4b

With the exception of areas of intraduct carcinoma mammography is not diagnostic of tumour histology. In radiographs of breast cancer fine calcification is a valuable diagnostic sign and commonly denotes areas of intraduct cancer (Black and Young, 1965; Levitan, Witten and Harrison, 1964; Sheppard, Crile and Strittmatter, 1962). We found that marked but not necessarily minimal calcification was associated with intraduct carcinoma. The fact that this relationship was absent in 6 of 17 radiographs may be explained by a failure to obtain the appropriate tissue for microscopic examination in lesions showing limited calcification. We found also that intraduct carcinoma may be present without calcification. Calcification was not definitely related to contour except that it was rare in smooth tumours. This confirms the findings of Ingleby and Gershon-Cohen.

Since it is rare for the whole of a breast tumour to be of intraduct type (Cutler, 1961; Bloom and Richardson, 1957) the presence of areas of fine calcification will not usually have a prognostic significance.

Lymphatic invasion is difficult to predict by mammography. The best correlation, as might be expected, was with extensive tumours. There was some relationship to trabecular abnormalities and a doubtful association with brush and blurred tumour margins. There is evidence, however, that when the radiological stromal changes are widespread and include extensive skin thickening, they may, in themselves, be of value as an unfavourable prognostic sign (Picard, 1962).

### CONCLUSIONS

(1) Mammograms provide an accurate overall representation of the majority of carcinomas, except when the breast is extensively involved with tumour.
(2) Mammography is of some value in detecting areas of intraduct carcinoma.
(3) Mammography is of no value in detecting other histological features.
(4) A gross tumour outline classification is described.

We wish to record our thanks to Mrs. Ursula Jones, British Empire Cancer Campaign technician, who prepared most of the paper sections.

We are indebted to Professor A. P. M. Forrest for continued help and encouragement throughout this project and to Professor K. T. Evans for access to the radiographs.

This work was supported by the British Empire Cancer Campaign for Research from whom one of us (H.J.S.) was in receipt of a whole-time grant, and by the Tenovus Fund for Cancer Research. It formed the basis of a paper given to the Surgical Research Society in July, 1966.

### REFERENCES

Black, J. W. and Young, B.—(1965) *Br. J. Radiol.*, **38**, 596.
Bloom, H. J. G. and Richardson, W. W.—(1957) *Br. J. Cancer*, **11**, 359.
Cutler, M.—(1961) 'Tumours of the Breast'. Philadelphia (Lippincott).
Egan, R. L.—(1960) *Radiology*, **75**, 894.
Gershon-Cohen, J. and Berger, S. M.—(1963) *Radiol. clin. N. Am.*, **1**, 115.
Gough, J. and Wentworth, J. E.—(1960) 'Recent Advances in Pathology'. 7th Edition. Edited by C. V. Harrison. London (Churchill), p. 80–86.

INGLEBY, H. AND GERSHON-COHEN, J.—(1960) 'Comparative Anatomy, Pathology and Roentgenology of the Breast'. Philadelphia (University of Pennsylvania Press) pp. 359–376.
LANE, N., GOKSEL, H., SALERNO, R. A. AND HAAGENSON, C. D.—(1961) *Ann. Surg.*, **153**, 483.
LEVITAN, L. H., WITTEN, D. M. AND HARRISON, E. G.—(1964) *Am. J. Roentgenol.*, **92**, 29.
PICARD, J. D.—(1962) *Acta Un. int. Cancr.*, **18**, 823.
SHEPPARD, T. J., CRILE, G. JR., AND STRITTMATTER, W. C.—(1962) *Radiology*, **78**, 967.
THOMSON, J. W. W.—(1963) *Proc. R. Soc. Med.*, **56**, 775.